# Case report of peritoneal carcinomatosis of plasma cell origin in a patient with newly diagnosed HIV: A terminal event

**DOI:** 10.1186/s12981-021-00369-5

**Published:** 2021-07-19

**Authors:** Syeda Sahra, Abdullah Jahangir, Muhammad Yasir Anwar, Allison Glaser, Ahmad Jahangir

**Affiliations:** 1 Department of Internal Medicine, Hofstra School of Medicine, Staten Island University Hospital, 475-Seaview Avenue, Staten Island, NY 10305 USA; 2 King Edward Medical University, Lahore, 54000 Pakistan

**Keywords:** AIDS, HIV, EBV, Plasma cell tumor, Peritoneal carcinomatosis

## Abstract

**Background:**

B-cell tumors and plasma cell malignancies have been identified in persons living with the human immunodeficiency virus (PLHIV). The literature review has revealed numerous reports of solitary plasmacytomas with metastasis in PLHIV.

**Case report:**

A young patient with no prior medical or surgical history presented with tumor lysis syndrome secondary to metastatic plasma cell Epstein-Baer virus (EBV) related malignancy with peritoneal carcinomatosis. The history and clinical picture promptly led to the diagnosis of HIV. The subsequent hospital course was dismal, and lifespan was cut short by multi-organ failure.

**Conclusion:**

This case is being reported to highlight the suspicion of HIV in patients presenting acutely with aggressive plasma cell malignancies.

## Introduction

Due to the advent of combination antiretroviral therapy (cART), leading to the effective management of HIV complications, the overall survival of persons living with human immunodeficiency virus (PLHIV) has increased [1]. The general incidence of acquired immunodeficiency syndrome (AIDS) defining malignancies has been decreasing significantly after the introduction of (cART) as well [2]. However, several non-AIDS-defining malignancies are rising due to concurrent exposure to other carcinogens and patient-related factors. The association of HIV with malignancies, in particular plasma cell tumors, is well established. Sporadic cases of plasma cell proliferation disorders have been reported in recent years. We present a case of a young male who was found to have an aggressive hematological malignancy on initial presentation, which was found to be a plasma cell neoplasm on lymph node biopsy. He was subsequently found to have advanced HIV infection. The case is being reported to suspect HIV is a widely metastatic plasma cell disorder along with other AIDS-defining malignancies (Sarcoma, non-Hodgkin lymphomas, and cervical cancer).

## Case presentation

A 33-year-old African American male with no prior medical or surgical history except for recent hemorrhoids presented to the emergency room of New York City in the second half of 2020 with three months of abdominal distention, nausea, non-bloody vomiting, and weight loss. He also reported pleuritic chest pain, which was localized to the lower chest bilaterally. A review of systems was positive for weight loss, constipation, and hematochezia. He denied any fevers, chills, sick contacts, recent travel history, COVID-19 exposure, skin rashes, excessive bleeding, or arthralgia.

The patient reported a recent episode of shingles around his eye and a fall without any loss of consciousness on further inquiry. His social history was significant for unprotected sex with multiple male partners. The patient denied intravenous drug use, smoking, alcohol, or illicit drug use. He was never diagnosed or treated for a sexually transmitted infection (STI). The patient was not following with his primary care doctor, and his family history was remarkable for his mother having Factor 5 Leiden mutation with an episode of pulmonary embolism.

The patient was hemodynamically stable on admission (blood pressure 126/82 mmHg, heart rate 70 beats per minute, respiratory rate 16 breaths per minute, temperature 96.8 F, saturating 95% on room air). On physical exam, the patient appeared chronically ill and cachectic with temporal wasting. A small painless, immobile, nodular mass was noted on the right side of the chest wall. The abdomen was distended, and bilateral lower extremity edema was noted. The patient was documented to weighed 67 kg on arrival with a BMI of 20.7 kg/m2 (baseline found to be 120 kg as per the patient).

**Fig. 1:**
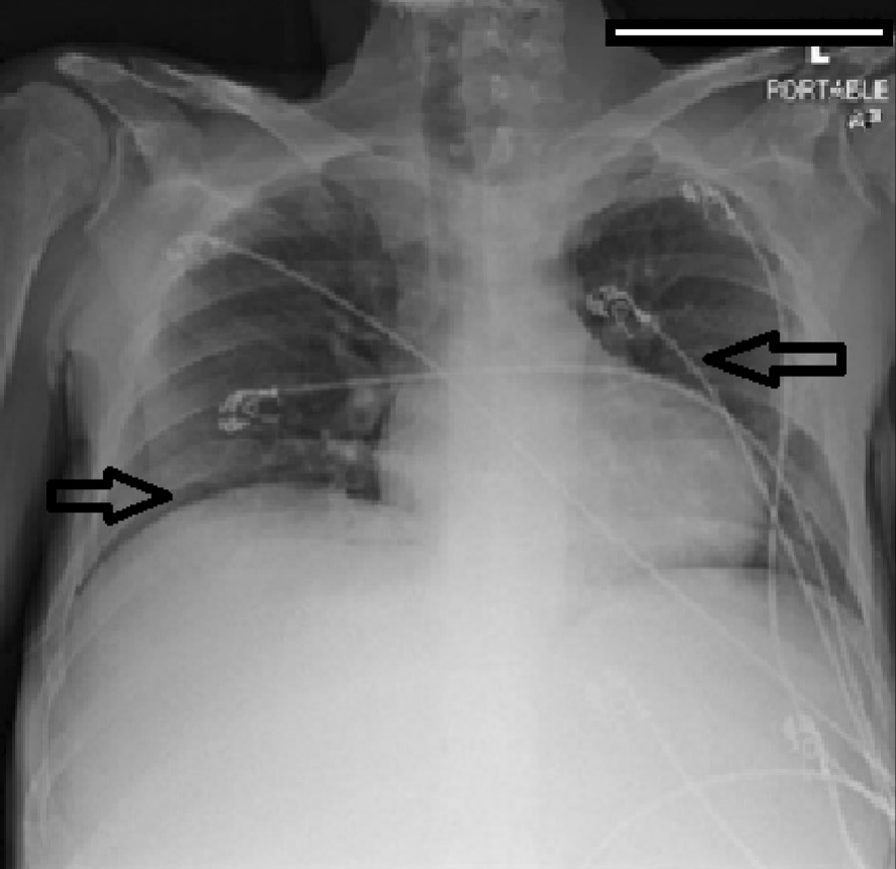
Chest x-ray showing trace bilateral pleural effusions and ill-defined erosive mass of left fourth and fifth rib

**Fig. 2:**
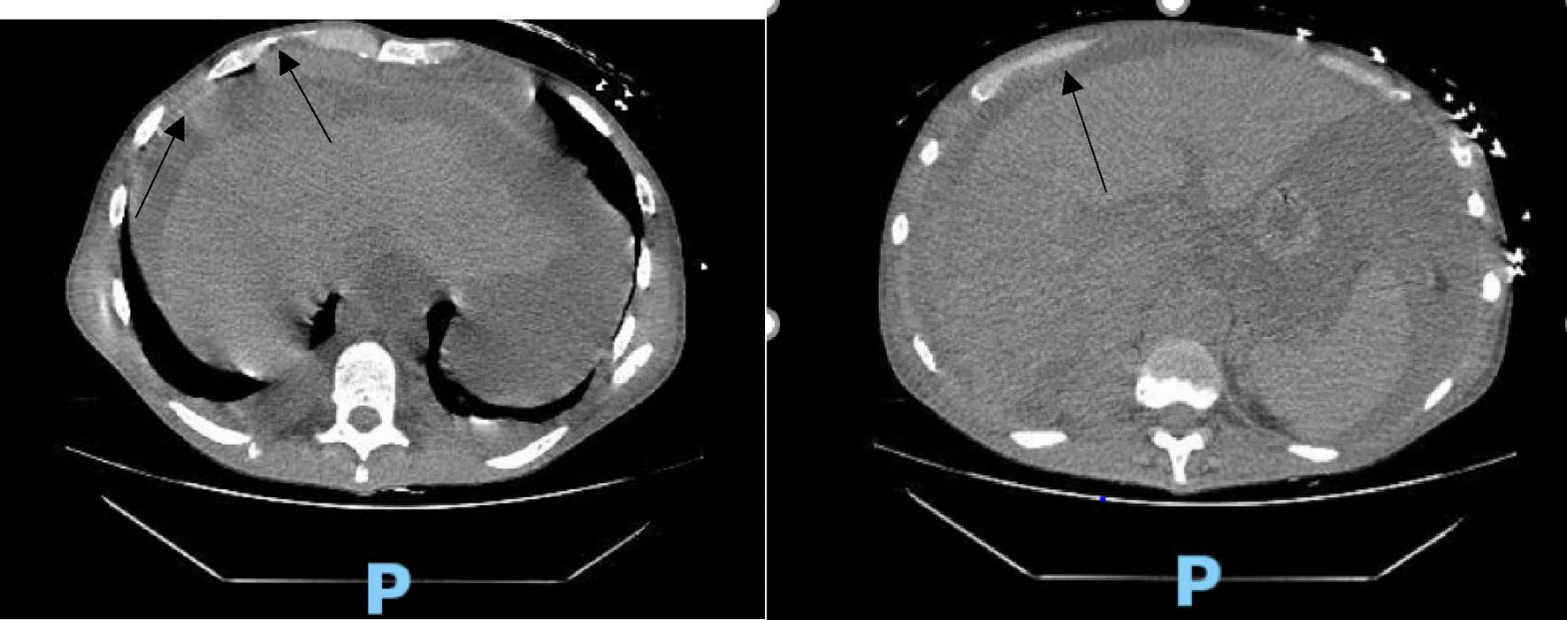
CT abdomen and chest showing heterogeneous liver, possible hepatic capsular implants, large volume ascites involving abdomen and pelvis and omental caking

**Fig. 3:**
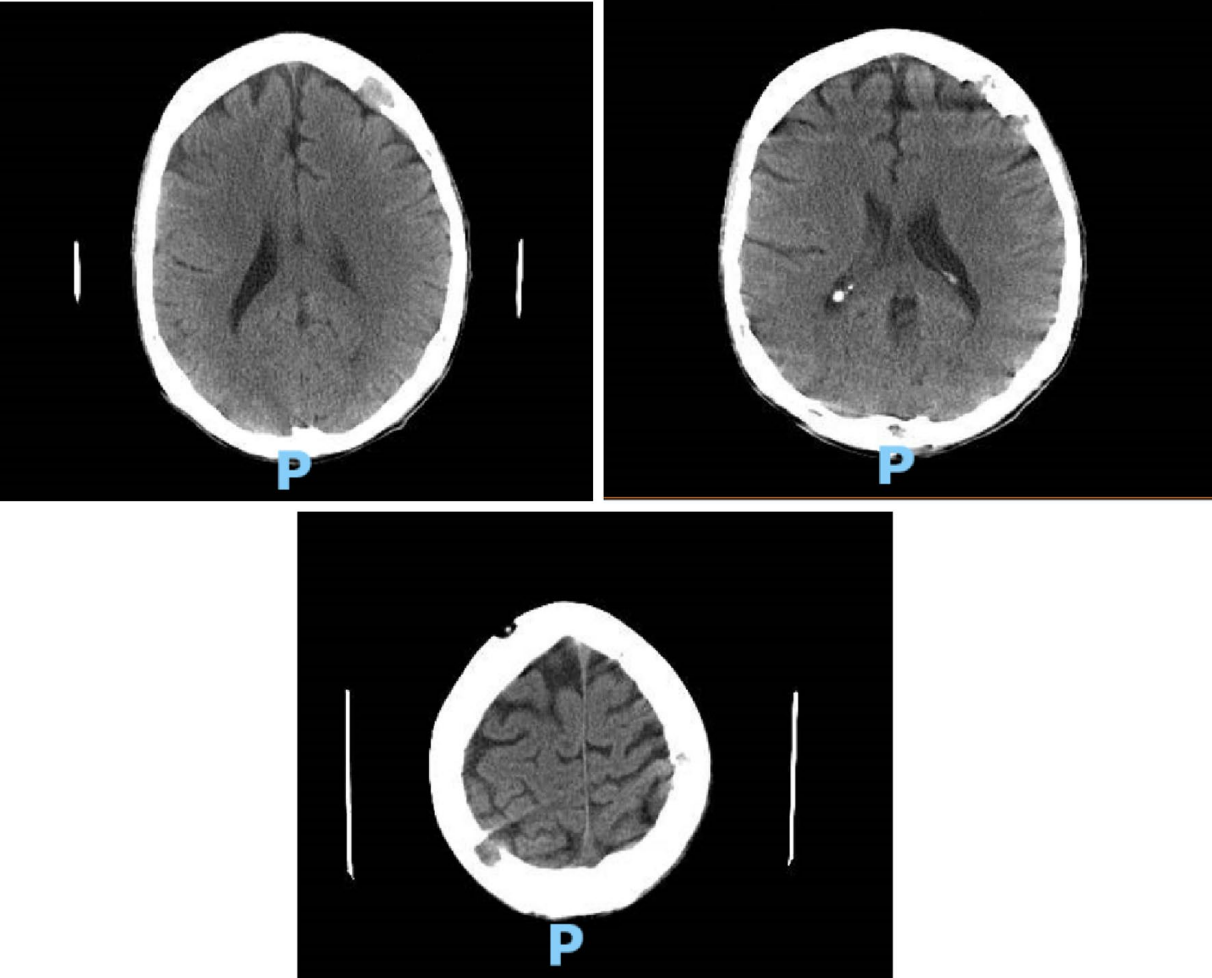
Multiple lytic lesions all over the extent of the calvarium

Hematology workup revealed mild leukocytosis (10.94 K/uL), normocytic anemia (hemoglobin 7.3 g/dL, mean corpuscular volume 94.4fL), and thrombocytosis (platelet count 901,000). Comprehensive metabolic panel was consistent with acute kidney injury (AKI) (creatinine 3 mg/dL, blood urea nitrogen 45 mg/dL—baseline unknown), tumor lysis syndrome (potassium 6.3 mmol/L, uric acid 21.7 mg/dL, phosphorus 5.6 mg/dL, and calcium 8 mg/dL). hyponatremia at 127 mmol/L, high anion gap metabolic acidosis (bicarbonate level 6, pH 7.23 on VBG, lactate 11.5), lactate dehydrogenase > 25,000 units/L, and elevated transaminases in a mixed pattern. Chest x-ray showed an ill-defined erosive mass of the left 4th to 5th lateral ribs, a thyroid mass, and trace pleural effusions (Fig. 1). Computerized tomography (CT) chest, abdomen, and pelvis had several significant findings pointing towards widespread malignancy. It showed a left supra-clavicular nodal mass measuring 5.4 × 4 × 3.6 cm, diffusely heterogeneous liver possibly reflecting metastatic disease, nodular soft tissue densities along with the hepatic capsule suspicious for hepatic capsular implants, large volume abdominopelvic ascites with extensive peritoneal soft tissue thickening/omental caking compatible with peritoneal carcinomatosis, mass-like thickening of the rectum, numerous lytic lesions involving the bilateral scapula, multiple ribs, bilateral proximal humerus, numerous vertebral bodies, pelvic bones, and proximal femur (Fig. 2). CT Head showed numerous diffuse lytic calvarial lesions concerning metastatic disease, but no mass effect was seen (Fig. 3).

The patient was transferred to the intensive care unit for medical optimization, further workup, and monitoring. His findings suggested an aggressive and rapidly progressing malignancy, possibly hematological in origin due to elevated LDH and labs consistent with tumor lysis syndrome. HIV antibody/antigen testing was ordered due to high-risk sexual behavior after obtaining consent from the patient, resulting in positive with a viral load of 271,782 copies/ml (Full T cell subset, CD4: 24%, CD4: 73/Ul). A paracentesis was performed (Table 1). Cytopathology showed malignant cells, and staining came positive for CD 138, CD 45 and negative for CK7, CK 20, AE1/AE3, cam5.2, CD3, CD20, CD 30, D2-40, calretinin, placental alkaline phosphatase (PLAP), CD117, PAX-8, CD68, CDX-2, Mart-1, prostate-specific antigen (PSA), prostatic acid phosphatase (PSAP), synaptophysin and chromogranin.

**Table 1:** Peritoneal fluid analysis

Colour-body fluid	Pink
Appearance-body fluid	Cloudy
Total nucleated cell count	15,421 u/L
Total red blood cell count	300,000 u/L
Monocyte/Macrophage count	59%
Fluid segmented granulocytes	17%
Body fluid lymphocytes	24%

Iron studies showed anemia of chronic inflammation. Hepatitis panel was reactive for Hepatitis C, QuantiFERON-TB Gold Plus (Qiagen) testing resulted as indeterminate. Toxoplasma IgG and IgM, EBV IgM, and early antigen were negative. EBV IgG and virus capsid antigen, nuclear antigen was positive, and the EBV viral load was 50,900 IU/mL. Cytomegalovirus (CMV) IgG also tested positive.

Tumor markers were obtained (Table 2). Paraproteinemia with increased M-spike was seen on SPEP. UPEP was found within normal limits (Table 3). Serum immunofixation showed elevated IFE lambda 6.48 mg/dL, elevated k/l ratio 2.98, elevated IFE kappa 19.29. Excisional biopsy of the right chest wall mass, a probable lymph node 1 × 2 cm, was performed for tissue diagnosis, which showed plasma cell neoplasm with pleomorphic morphology, focal necrosis, and high ki-67. Atypical cells were reported as plasmacytoid with eosinophilic cytoplasm, which was pleomorphic and large with a multinuclear, mixed population of kappa light chain restricted plasma cells.

**Table 2:** Tumor markers

Tumour Marker	Value
Carcinoembryonic antigen	1.1 ng/mL
Alpha fetoprotein	2.3 ng/mL
Cancer antigen GI Ca 19–9	61 U/mL (elevated)

**Table 3:** Serum protein electrophoresis

Protein electrophoresis serum	Value with normal range in parenthesis
Protein total, serum	5.9 (6–8.3 g/dL)
Albumin, serum	1.8 (3.6–5.5 g/dL)
Alpha 1	0.4 (0.1–0.4 g/dL)
Alpha 2	0.7 (0.5–1 g/dL)
Beta globulin	2 (0.5–1 g/dL)
Gamma globulin	1 (0.6–1.6 g/dL)
Albumin/globulin ratio	0.4 (Ratio)
Serum protein electrophoresis interpretation	Beta-migrating paraprotein identified
M-spike	1.3 (undetectable)

The patient responded to volume resuscitation initially but remained tachycardiac and hypotensive, further complicated by acute respiratory failure. D-dimer was elevated; pulmonary embolism could not be ruled out considering the history of Factor V mutation in his mother. CT angiogram of the chest was not performed given AKI, and he could not be started on anticoagulation owing to low hemoglobin and hematochezia. Echocardiogram was negative for right heart strain. The lower extremity venous duplex was negative for any deep venous thrombosis.

The patient continued to worsen over 72 h and was intubated on day 4 of hospitalization, requiring vasopressor support and renal replacement therapy. The medical team discussed goals of care and advanced directives with the family, who expressed their wishes not to resuscitate the patient in the event of cardiac arrest. The patient expired on day 5 of hospitalization secondary to cardiopulmonary arrest.

## Discussion

HIV infection complicated by immunosuppressed state defined as AIDS predisposes individuals to malignancies, commonly B cell lymphomas [3–5]. A handful of malignancies are categorized as AIDS-defining malignancies per the world health organization (WHO) classification to emphasize the causation and highly suspect underlying HIV infection if these malignancies are reported [6]. Plasma cell malignancies in PLHIV are rare but have been reported since the 1980s, including the rare plasmablastic lymphomas. The incidence has been on the rise, especially in cases with coinfection with EBV, a known oncogenic virus and causes unchecked proliferation of B cells. Most plasma cell proliferation is associated with light chain immunoglobulins. Some cases are observed to be highly responsive to cART if caught earlier in the timeline of malignant transformation[7]. Paraproteinemia has been reported in the cohort of PLHIV, pointing towards increased plasma cell proliferation in these patients [8–13]. Multiple myeloma has been seen in HIV-positive patients from a broad spectrum of presentations, from having chronic lymphadenopathy to multiple extramedullary plasmacytomas and osteolytic bone lesions.

The association of plasmacytomas with HIV has been well established. HIV-associated plasmacytomas have been seen in extramedullary sites as well as rare primary sites, including mesentery [14], ureter [15], and laryngeal wall [16], and also as a mass in the kidney [17]. Plasma cell disorders in such immunosuppressed populations are often severe with a noticeably short aggressive clinical course and in a younger patient population compared to the typical age group affected by plasma cell malignancies.

A case of plasmablastic lymphoma with a similar case presentation as ours was reported, including widespread malignancy involving the peritoneal cavity as well, but that particular patient was immunocompetent and tested negative for HIV [18]. This case, and the existing literature, highlight the importance of considering a diagnosis of HIV infection in the setting of plasma cell malignancies.

## Conclusion

About our case presented, all this evidence-based data points out that HIV, especially when undiagnosed with an exceedingly high viral load, compounded by oncogenic viral coinfections, predisposes an individual to plasma cell malignancies. The manifestation has been increasing since the initial publication in 1983 and has not been incidental. The presence of multiple myeloma, paraproteinemia, widespread aggressive malignancies should prompt an investigation to rule out HIV infection. Timely diagnosis and treatment in younger populations might prevent the essentially terminal prognosis.

## Data Availability

Relevant images and tables are reported in the manuscript and in attached files as tables and figures without any identifying information. Datasets generated or analyzed during the current study are attached and accessible on request.
